# Workflow Challenges of Enterprise Imaging: HIMSS-SIIM Collaborative White Paper

**DOI:** 10.1007/s10278-016-9897-6

**Published:** 2016-08-15

**Authors:** Alexander J. Towbin, Christopher J. Roth, Mark Bronkalla, Dawn Cram

**Affiliations:** 1 Department of Radiology, Cincinnati Children’s Hospital, 3333 Burnet Avenue, MLC 5013, Cincinnati, OH 45229 USA; 2 Duke Health Technology Solutions, Hock Plaza, 2424 Erwin Road, Durham, NC 27705 USA; 3 Department of Radiology, Duke University Hospital, 2301 Erwin Road, Box 3808, Durham, NC 27710 USA; 4 Merge Healthcare, an IBM Company, 900 Walnut Ridge Drive, Hartland, WI 53029 USA; 5 UHealth Information Technology, University of Miami, 1425 N.W. 10th Avenue, Miami, FL 33136 USA

**Keywords:** Enterprise PACS, Digital imaging and communications in medicine (DICOM), Clinical workflow

## Abstract

With the advent of digital cameras, there has been an explosion in the number of medical specialties using images to diagnose or document disease and guide interventions. In many specialties, these images are not added to the patient’s electronic medical record and are not distributed so that other providers caring for the patient can view them. As hospitals begin to develop enterprise imaging strategies, they have found that there are multiple challenges preventing the implementation of systems to manage image capture, image upload, and image management. This HIMSS-SIIM white paper will describe the key workflow challenges related to enterprise imaging and offer suggestions for potential solutions to these challenges.

## Introduction

Health care providers use images to help diagnose disease, document abnormalities or interventions, and guide treatment for their patients [1–17]. Together, these images help to tell the story of each patient’s clinical journey. Unfortunately, the majority of images are not visible to the team of doctors, nurses, therapists, technologists, and other clinicians caring for a patient. This is true for a number of reasons, most importantly including a lack of systems and workflows required to acquire, upload, and view images. While historically, the radiology and cardiology services have done a good job creating automated workflows for image acquisition and information systems for image distribution, these practices have not been adopted by other services in the hospital. Today, the majority of services use some form of imaging in their practice [1, 2, 4, 5, 7–13, 16, 18–20].

As images have increased in importance, hospitals have struggled to effectively store, display, and distribute these images throughout the enterprise. This has remained a struggle because of inefficient workflow and incomplete solutions. The purposes of this HIMSS-SIIM white paper are to describe the key workflow challenges related to enterprise imaging, offer suggestions for potential solutions, and identify areas where there is an opportunity for the health care information technology community to find an innovative solution. Specifically, this paper will address clinical and informatics workflow challenges related to clinical efficiency, accurate patient and image identification, image acquisition and image quality, and end user image consumption. In addition, this white paper will identify several potential legal concerns.

### Problem #1: Workflow

Each specialty acquires and uses images differently. In radiology, the image acquisition process begins with an order placed by a referring physician. Once the order is placed, it is transferred to the radiology information system (RIS). The RIS uses the information contained within the order to create a worklist on the imaging modality. Technologists select the patient from the worklist, ensuring that all demographic and order information is correct. After the images are obtained, they are sent to the picture archiving and communication system (PACS) for storage and viewing. Even though this workflow is elegant, it is not able to be generalized within the hospital.

Radiology departments require orders to perform an imaging study. This requirement is in place because historically radiologists are not the physician working up a disease process. Instead, other clinicians evaluate the patient, create a differential diagnosis, and then use radiologic imaging to refine their differential diagnosis. In this scenario, the ordering physician asks the radiology department to perform a specific study. Because the ordering clinician’s evaluation is separate in time and location from the radiology department, the order acts as a form of communication between the two practices.

The same requirement for an order does not always exist in other divisions or departments of the hospital. For example, a dermatologist may see a patient in clinic and take a photograph in order to document a skin lesion. Because the dermatologist is the one taking the photograph, he or she does not need an order to perform this imaging procedure. It would be inefficient and ineffective if the dermatologist was required to place an order in the same manner as if he or she was placing an order for radiologic imaging.

In the modern radiology department however, orders are used for more than just communication of which study needs to be performed. Today, the order helps to drive an automated workflow by creating a unique study identifier and a PACS worklist of patient studies needing review. If an order is not needed for imaging in other specialties, we, as an industry, must identify other methods for creating a unique identifier and patient worklist. Currently, hospitals have taken one of two approaches to solve this problem: they either continue to use an order-based workflow or they use the encounter to drive the workflow. A detailed discussion of the merits and shortcomings of each approach presented in the whitepaper entitled “Orders Versus Encounters Based Image Capture: Implications Pre- and Post-Procedure Workflow, Technical and Build Capabilities, Resulting, Analytics and Revenue Capture.”

### Problem #2: Patient Identification

Correct patient identification is imperative. The correct images must be placed within the correct patient’s medical record every time. As such, all images must include patient identification. In digital imaging and communications in medicine (DICOM) imaging, this identification is automatically applied with an order selected from the modality worklist supplying the necessary metadata, as described above. The DICOM modality worklist can be created by various sources including the electronic medical record (EMR) system, the RIS, the PACS, and the enterprise archive [21]. The system that creates the DICOM modality worklist sends key patient demographic information to the imaging modality. This demographic information usually comes from the order and includes the patient’s name, medical record number, date of birth, sex, and the procedure name. The imager can select the patient from the worklist in a number of ways including focused query, direct selection, or barcode scanning.

In non-DICOM imaging, an automated solution must be identified to correctly identify patients. Potential solutions include workflow reminders, adding patient identifying information to every image or create a new modality worklist. These scenarios are described in more detail below with their associated advantages and disadvantages.

#### Workflow Reminders

Some practices currently use workflow reminders in order to correctly identify patient images. These systems include practices such as bracketing medical photographs with patient identifiers (taking a picture of a patient intake form before and after a series of medical photographs) and photographing one patient at a time on reusable media. While these systems can be effective, they rely on humans to remember to perform the correct procedure every time. Because there are no reminders and no fail safes, errors can occur and lead to patient misidentification. In addition, newer cameras, including mobile devices, may not have an option for reusable media.

#### Include Patient Identifying Information in Every Image

This is an easy, low-tech solution whereby an identifier such as a sticker or barcode is placed on or near the patient and included in the photograph. While this is an effective solution and can prevent misidentification at a later date, there are still several disadvantages of this practice. First, the identifying data always resides with the images. While there are definite benefits of this, we have learned through DICOM-based modalities such as ultrasound that “burned in” data is not ideal. This is particularly true when the images need to be anonymized, either for research or for medical conferences. The second disadvantage of this practice is that it cannot be standard throughout the hospital. There are many instances where a patient sticker, barcode, or another identifier cannot be placed. Examples include intraoperative photographs where sterility must be maintained and photographs of internal structures such as with endoscopy. Finally, even though stickers are relatively inexpensive, they add cost to the organization.

#### Create a New Modality Worklist

There are several ways a modality worklist equivalent can be created. Some institutions use a health level 7 (HL7) admission, discharge, transfer (ADT) interface to mimic a modality worklist based on encounters. In this instance, the interface engine is used to perform an ADT to order transformation, effectively mimicking an order-based DICOM modality worklist to devices. Since DICOM modality worklists do not support visit entries, the order transformation allows the devices to select an actual order. The end result of this transformation is that the imager is able to select patient demographic information in a manner similar to how DICOM modality worklists are used. Images can be associated with the modality worklist entry either before or after imaging is performed.

Typically, the modality worklist can be accessed BEFORE imaging by “connected” or “smart” devices. Examples of these devices include smartphones, tablets, or other networked imaging devices. In this scenario, the imager could select the patient from the worklist before taking an image. Once the imaging is complete, the image is sent to the imaging archive along with patient demographic information. While this workflow may be ideal, it may limit hospital use of certain consumer technology such as digital cameras.

Another option would be to apply the demographic information to the image AFTER it is obtained. In this case, because the information is applied after the images are obtained, the same workflow considerations related to patient identification that are described above must be accounted for. In this scenario, the correctly identified images could be uploaded from within the patient context within the electronic medical record. In addition, this option requires providers to perform the additional steps required to upload images at a separate time point. There is a risk that image upload will not be performed by busy providers unless it is tied to image acquisition.

It is likely that a number of these scenarios may be used to upload data. If that occurs, it may be useful to develop a confidence score for the reliability of the data. System-entered data (such as data generated from a modality worklist) would have a higher confidence score compared to manually entered data.

### Problem #3: Information Needed in an Image

If images are used to diagnose an abnormality or to help provide an objective measure for long-term follow-up, the images must have certain qualities to allow for further study and comparison. While this has been addressed by many DICOM modalities, there is a need for all images to possess certain qualities. Some of the qualities we discussed as well as potential solutions are listed below:

#### Standard Measurements

Length and width measurements are key criteria in many disease processes. Simple measurements allow health care providers to quickly assess how a disease process changes over time [4, 5, 12, 18, 22]. The ability to perform a measurement is a key feature of DICOM-based imaging. For example, in radiologic images, one can measure the diameter of a tumor as the exact size of each pixel is known. In addition, certain DICOM-based studies, such as Doppler waveforms on echocardiography, allow the user to measure time. In the cases where the size information is not known, DICOM images can be calibrated using an imaged ruler so that measurements can still be obtained. This model should prove to be useful in non-DICOM imaging. Because of this, best practice might be that rulers be included in all images that do not have inherent size information. The standard use of a ruler helps to mitigate against differences in the appearance of a lesion due to zoom factor and the distance between the camera and the lesion.

#### Color standardization

Standardizing color values has not been addressed in the traditional imaging services. This is because most radiologic and cardiologic are imaged using shades of gray. When color is used, such as in nuclear medicine or color Doppler imaging, the hue is not as important as the intensity. This principle is not true in other specialties. In specialties such as dermatology, pathology, and wound care, creating reproducible colors is important [4, 5, 9, 15, 17, 23, 24]. However, reproducible color is challenging due to differences in lighting (incandescent versus fluorescent versus natural), shadowing, and camera settings.

Standardizing color may be difficult. Perhaps the best method for standardization is through the use of a medical photographer. A medical photographer can obtain images in a photo studio, controlling the light, background, and even camera settings. By having a standard protocol for imaging, the differences at each imaging time point are lessened [2, 4, 25]. While the use of medical photographers may be possible in an ambulatory setting, it is unlikely that this practice will translate to the inpatient setting, the operating room, or the emergency department. Therefore, other strategies for standardizing color should be considered.

Other potential options include incorporating a color wheel, grayscale, or white card within the photograph. Once photographed, a computer could then automatically calibrate the color to the accepted standard. There are several disadvantages of this system. First, each of these solutions must be printed, either on paper or plastic. In order to ensure consistency of the process, high-quality printers would have to be used. This would add cost to the hospital. Like printed identifiers, these printed color wheels could only be used in certain settings. However, both the cost and location issues could be mitigated by identifying the specialties where reproducible color is needed. The remainder of specialties could produce images without color calibration. Additionally, including items such as a color wheel, patient identification sticker, and ruler all add to the space required in the image. In order to account for this space, the image field of view may have to be increased thus decreasing the available space for the clinical region of interest.

It is also important to understand the color correction features of modern digital cameras and the potential effect that they may have on medical photographs [4, 5, 17, 24–26]. Consumer camera manufacturers have developed a number of features that modify images automatically. While features such as red-eye removal, blemish correction, and image filters enhance personal photographs, they may mask medical conditions and provide unpredictable and unreproducible content for health care providers. In general, these features should be turned off when possible.

#### Standard Patient Positioning

Standard positioning is crucial to many of the traditional imaging services. Because radiologists and cardiologists identify abnormalities based on pattern recognition, they rely on a standard appearance of body parts. This standard positioning allows them to quickly distinguish normal from abnormal. In specialties where imaging is not used for diagnosis, positioning may be less important. The major benefits of standard positioning in these specialties include the ability to correlate abnormalities with anatomic landmarks and the ability to document an abnormality in the same way at multiple time points [2, 4, 25]. Even if standard positioning is not needed in many specialties, it may be useful to annotate the location or patient positioning on the image for future reference.

### Problem #4: Reporting

Images are worth a thousand words, but only if you understand the context of the image. A person can describe a photograph of a landscape in detail because he or she understands what the photograph is showing, even if he or she has never seen the location. The same is not true for medical imaging. While a medical provider can describe portions of an image, specialists are needed to provide the exquisite detail in describing the image. Therefore, it is crucial to be able to link the text describing an image or an encounter with each image. For the purpose of this discussion, the description of the image will be called a report. However, it should be noted that in this situation, a “report” refers to any text that gives the image context. Thus, a report for a dermatology image may be a referral letter, and a report for an operative photo may be an operative note. There are multiple challenges related to reporting in this scenario.

First, the report serves different purposes for different specialties. In radiology and cardiology, the report is used to provide an interpretation of the images. In other specialties such as dermatology, the report is used to describe the entire visit. Because of these differences, reports reside in different locations in the electronic medical record. In order to prevent the proliferation of notes in the electronic medical record, it is our opinion that these reports should not be duplicated. Therefore, each report should reside in its current location (i.e., a dermatology letter should not be duplicated or moved to an imaging tab or results tab).

In addition, certain imaging studies may have multiple reports. In cardiac catheterization, for example, the report may be both the operative note and the detailed functional data. In these instances, both reports give the image context and should be associated with the images. In current electronic medical record systems and enterprise viewing systems, there is no method for assigning multiple reports to one imaging study. This limitation can be mitigated within a viewer by treating one or both reports as an image.

Third, reports are created at different stages in image acquisition. In radiology, the images always precede the report because the radiology report is an interpretation of the images. However, in dermatology, the images serve to document the finding. Thus, the images may be obtained after the report has already been dictated. Electronic medical record systems must allow users to create the association between images and reports at any stage of the workflow.

Finally, images must be associated with reports in a bi-directional manner. This means that there must be a way to view images (whether via a link or direct visualization) within the report in the electronic medical record AND a method to view the report within the enterprise viewer and a link to launch the encounter in the electronic medical record. Associating reports and images in both systems allows medical providers to review patient information according to their preferred workflow. A pediatrician may read an operative report and click a link to view the photographs from a surgery. While she is viewing the operative images, she may see that pathology images are also present. In this scenario, the pediatrician should not have to click back to the electronic medical record system to read the pathology report. Instead, she should be able to click a report button within the viewer to read the pathology report. Currently, it is difficult to create the association between images and a report, particularly if there is no order for the images. As encounter-based imaging becomes a standard workflow, the electronic medical record systems must enable this functionality.

### Problem #5: Metadata

Reports are not the only information to give image context, metadata also serves this purpose. In DICOM-based imaging, metadata is applied at the patient, study, series, and image level [27]. For the following discussion, we will focus on study-level information as it provides the basic information needed to classify an imaging study. However, it is likely that series and image level metadata will need to be modified for enterprise imaging. This information is crucial for driving display protocols, comparison studies, and searching.

#### Body Part

In the setting of enterprise imaging, certain pieces of information increase in importance. Perhaps the most important piece of information for searching and sorting across specialties is the body part imaged. For example, a patient involved in a car accident may sustain a right knee injury. As part of his or her care, a photograph of injury is obtained in the emergency room. The patient then travels to radiology where an X-ray and MRI are obtained. Finally, the patient is brought to the operating room where he or she undergoes arthroscopic surgery. The images from all of these studies are related and should act (or at least have the potential to act) as relevant comparisons.

Radiology has a certain structure based on DICOM standard body parts [27]. While this structure works for radiology, it does not work in the setting of enterprise imaging. In some cases, the naming structure is too specific while in others, it is not specific enough. An example of a term that is too specific is the humerus. While this term makes sense in for an X-ray of the upper arm, it does not make sense for a picture of the skin of the same location. On the other hand, the term abdomen is used when, for surgical or pathologic images, this is not specific enough. In these specialties, it is important to refer to the specific organ being imaged, such as the liver or pancreas.

We recommend that a standard ontology be adopted for the purpose of body part mapping. The selected ontology should allow for synonyms and have a relational structure so that body parts can be considered parent and children terms. Thus, humerus and upper arm are considered to be synonymous. At the same time, humerus, forearm, hand, and fingers are all part of the upper extremity.

#### Procedure Description

In radiology, study-level metadata is applied directly from the order. Perhaps the most important metadata applied to a radiology study is the procedure description. This one field may contain information relating to up to four variables. For example, the procedure description “RAD Hand 2-3V Right” tells the provider that the imaging study is (1) a radiograph (RAD) containing (2) 2–3 views of the (3) right (4) hand.

While the procedure description has worked in radiology, there are several limitations in the setting of enterprise imaging. First, there is no standard way to create a procedure description. This is best exemplified in the naming of a chest X-ray. Depending on the setting, this procedure can be named Rad Chest, Chest X-ray, Chest XR, or CXR among others. The American College of Radiology has identified this problem in creating the dose index registry [28, 29].

This problem is compounded by sites creating study-specific orders. For example, an MR enterography is a special type of MRI of the abdomen and pelvis performed to diagnose and monitor inflammatory bowel disease. Creating a study-specific order is useful for several reasons. It allows health care providers to order a specific study for a specific indication. A radiologist can then create a structured report based on this specific order. Finally, with study-specific orders, practices can better understand the ordering patterns of their providers. While there are benefits of this practice, the biggest disadvantage is the explosion of orderable procedures within the electronic medical record. The ACR has addressed this issue by asking providers to map their procedures to standard procedures identified in the RadLex playbook [28–31].

As we move to enterprise imaging, it is possible that certain imaging workflows are encounter based, not order-based. In this scenario, there must be an efficient way to create a standard procedure description. Each procedure description should contain at least two pieces of information, the study type and the body part imaged including the side of the body. The study type helps to differentiate similar studies within the same department. This allows an ultraviolet photograph of the face be different from a visible light photograph of the face [19, 32]. Given the complexity of searching and creating hanging protocols in an enterprise viewer, it may make sense for the body part to be duplicated in a separate field.

#### Department

In many current radiology and cardiology PACS, the department DICOM field is of limited value. As all of the images in the hospital come together, this field becomes crucial. It is important that a dermatologist can find all of the dermatology images quickly and distinguish them from radiologic images of the same body part. Image viewers must be able to allow users to search and sort based on this field.

Like the body part field, this field should be standardized. The biggest challenge in standardizing the department metadata is identifying the correct level of granularity for each specialty. While in most instances, it makes sense to use named divisions such as cardiology and gastroenterology in the department field, in other specialties such as radiology, this may not make sense.

#### Imaging Source

Because traditional imaging modalities represent a significant capital expenditure, medical imaging has been performed exclusively by medical practices. However, this paradigm is shifting. As digital cameras become increasingly prevalent, many new imaging providers exist. This includes patients taking photographs of themselves to share with a health care provider. As patients begin to upload their own images to an electronic medical record or enterprise imaging archive, there will likely be a need to be able to tag studies as either patient obtained or provider obtained. The difference may be important for quality assurance purposes, liability concerns, and even reporting related to meaningful use.

### Problem #6: Legal Concerns

As enterprise imaging matures, there are several potential legal concerns that should be addressed [20, 33–35]. The following is not meant to be an exhaustive discussion of the topic, rather it is meant to highlight key topics. Each of the following topics will have to take local law and restrictions into account.

#### Patient Privacy and Access Control

There are unique privacy concerns that exist with certain types of photographs. It is recognized that institutions may choose to limit access to certain types of images such as images related to sexual assault or child abuse. In these scenarios, there are several options vendors may choose to allow institutions to manage permission to view these images. Potential options are listed below.

First, electronic medical record systems may be used to manage the permissions to view images. In this case, permissions may need to be applied for all images related to a specific department such as plastic surgery or to images related to a specific procedure description such as sexual abuse imaging from the emergency department. In each scenario, it is important that users have access to view the report within the electronic medical record, but not view the images related to the report. Because users could access images either via the electronic medical record or the image viewer, users should be restricted in both systems. However, ideally, only one system is required to manage permissions.

A second potential mechanism for sensitive images may be to request permission on a case-by-case basis. If vendors and institutions select this option, a user would have to select a reason why he or she needs to view the restricted images. These reasons would be recorded and could later be audited. Both the viewer and electronic medical record system would identify and flag users who frequently view restricted images. These users could then be audited to ensure that they are viewing restricted images in line with their patient care duties. Ideally, a permissions-based system could be coupled with an override capability so that certain departments such as plastic surgery are not over-burdened in an attempt to view images relevant to their practice.

Another challenge related to restricted images is image sharing. In the setting where sensitive images are considered part of the medical record, hospitals need to determine mechanisms for the types of images they are willing to share and the permission required to share these images. It is possible that hospitals choose to flag certain images and require a separate permission/consent to share these images with other institutions or providers.

#### Maintaining Images

In the analog era, images were printed on physical media. Because there was a direct cost associated with each printed image (both for film and for paper), fewer images were obtained and printed. In the digital era, most of the direct costs have been removed and images exist as a collection of bits and bytes on a server. While there are costs associated with storage, this cost is often small per megabyte and removed from the imager.

In the consumer market, we have devices that that can take and store thousands of images. Many of us now have archives of thousands of personal digital photographs. As such, we have an expectation of keeping everything. In the medical world, this may not be needed or appropriate. While this decision will likely play out in individual hospitals, through legal case precedent, and possibly legislation, it is important to think of this concept ahead of time.

Several states have legislated (or mandate through legal precedent) that radiology images be maintained for a certain amount of time. While most hospitals maintain the radiology images obtained at their institution, they do not archive or keep all images across the enterprise. In fact, each department or division maintains their images differently. In many cases, this even varies within a department. For example, in radiology, some images or series are rejected at the modality (such as due to motion) and never sent to the archive. In ultrasound, the technologist may select the images he or she wants to capture during live imaging. In most cases, the entire study is not recorded. Other specialties have a similar workflow. Specialists may review all of the images at the end of a procedure and select the key images that document the finding or the procedure. The other lower quality or less representative images may be discarded.

#### Video

The use of video as a method of documentation will likely explode in the coming years. This new data source has the potential to cause a significant increase in the size of the imaging archive. As video is obtained, hospitals may choose to place a limit on the length of the video or the types of exams or procedures that are recorded. It should be noted that video editing may be overly burdensome. Technology that allows providers to prospectively and retrospectively select segments to record should be encouraged. For example, an endoscopist should be able to push a button or press a foot pedal to begin recording. In addition, he or she should also be able to press another button or foot pedal to capture the previous 1–3 min of video. Finally, the process of video editing is not without risk. Hospitals will have to determine who is allowed to edit the video, which elements are required to sufficiently document each procedure, and how procedures or examinations are selected for recording.

#### Image Fidelity

As cameras improve, the file size for each image is increasing in size. This has the potential to cause a significant increase in the size of the imaging archive. In radiologic imaging, the U.S. Food and Drug Administration (FDA) requires that when an image is displayed, it be labeled with a message stating if irreversible compression has been applied and with approximately what compression ratio [36]. In addition, the FDA states in the Mammography Quality Standards Act that irreversible compression of digital mammograms for retention, transmission, or final Interpretation is not allowed, although irreversibly compressed images may be used for images from prior studies [37]. The ACR-AAPM-SIIM technical standard for the electronic practice of medical imaging makes no statement on the type or amount of compression that is allowed for a medical image to be called “diagnostic [34].” The European Society of Radiology suggests that image compression may be acceptable for certain specialties or in certain imaging types such as teleradiology or in long-term image storage and unacceptable in other specialties or for other imaging types such as in mammography, radiation oncology, or surgical navigation [38]. It thus follows that allowing for lossy images in certain scenarios or with certain file types may be allowed. If vendors enable multiple methods of image compression, it may allow to improve viewer performance and reduce storage requirements.

If lossy images are allowed, there should be a balance between the amount of compression and the subsequent image quality. With photographic images, the degree of lossiness is not normally expressed in a compression ratio but a standard specific quality factor. For example, JPEG lossy with a quality factor of 85 or better is seen as “high quality” or “visually lossless” to most people. If medical photographs were saved using a high-quality factor, significant space savings may be achieved without apparent loss of image quality.

#### File Format

File format is closely related to image fidelity. Some file formats, like joint photographic experts group (JPEG), can be considered lossy, while others, like tagged image format (TIF) or RAW formats, are considered lossless. It should be noted that lossless file formats such as RAW may not be standard across vendors. As hospitals move toward enterprise imaging, they may choose to select lossy formats for standard image storage. This decision may be based on several factors including file size, industry standards, the ubiquity of format, and the capabilities of enterprise image viewers. In addition, files can be viewed natively or wrapped using an industry standard format such as DICOM. The discussion of these options extends beyond the scope of this white paper. Further information on the subject can be found in the white paper entitled Technical Challenges of Enterprise Imaging.

### Problem #7: Mobile Devices

It is clear that providers are currently using their devices to capture images, videos, and sounds from their patients [1, 3, 7, 9, 33, 39]. In many instances, this practice raises concerns related to patient privacy. Mobile devices, such as smartphones, are designed to allow users to take and share photographs. Many people have hundreds of photographs stored on their phone and personal cloud solutions. For health care providers, this can include patient information. While using a personal mobile device is not ideal for patient care in the current setting, there are important reasons providers use their device, including multifactor authentication, review and creation of documents within a mobile electronic medical record application, and consultation via text message. Thus, it is important that hospital and vendors enable providers to use their devices in a manner that protects patients and meets clinical needs. In addition, because smartphones and tablets are Web-enabled, they also offer many advantages to other image capture devices. Vendors can take advantage of smart devices to develop applications for image capture. As applications are developed, the following features should be encouraged.

1. Ability to query the electronic medical record or broker for patient demographics.
2. Ability to scan a barcode or radio frequency identification (RFID) on a patient wristband.
3. Ability to take a photograph, photographs, or video.
4. Ability to display, select, discard, edit, and annotate photos and videos.
5. Ability to record sound.
6. Ability to associate metadata based on body part, side of body, and department.
7. Ability to send images to an archive.
8. Ability to compose an EHR note giving context to the images.
9. Ability to recognize the specific user (login required).
10. Ability to send colleagues a message within the system for consultation.
11. Ability to record the consultant’s response and deliver it to the electronic medical record.
12. Images must not store to the devices digital photo or video library (either for originating device or consultant’s device).
13. Patient demographics must not store to a local device or application database.
14. Ability to recognize device and ensure that it meets minimum standards for image quality.
15. Ability to provide a secure connection to the enterprise imaging archive for transmission of images and worklist queries.
16. Compatible with all major operating systems.
17. Ability to place an order related to the image.

While some enterprises or specialties may choose to embrace mobile applications as a method of documenting patient care or physical exam findings, other enterprises or specialties may choose to rely on conventional digital cameras. It should be noted that there are challenges related to these devices as well. While some smart/Web-enabled digital single-lens reflex cameras exist, they are not the norm. If cameras are selected that are not Web-enabled, providers may have more steps in the image acquisition and upload process. Many of these challenges are further described in the patient identification and metadata sections above. In addition to these challenges, traditional digital single-lens reflex cameras can add cost and risk of theft.

## Conclusion

Enterprise imaging is becoming a mainstream expectation of patients, hospitals, and health care providers. As the industry begins to address this need, many workflow and solution challenges remain. This HIMSS-SIIM white paper discusses seven key challenges related to enterprise imaging that need to be addressed before the practice can be considered mature. Each challenge will involve the cooperation of large societies, such as HIMSS and SIIM, standards bodies such as integrating the health care enterprise (IHE) and DICOM, vendors, hospitals, and health care providers. While some hospitals are moving forward and solving these challenges locally, the industry must quickly adapt standards to enable the practice of enterprise imaging. If done correctly, these standards will help enterprise imaging to serve as a pillar of the complete electronic medical record.
